# The long non-coding RNA Snhg3 is essential for mouse embryonic stem cell self-renewal and pluripotency

**DOI:** 10.1186/s13287-019-1270-5

**Published:** 2019-05-31

**Authors:** Weisi Lu, Jianping Yu, Fengtao Shi, Jianing Zhang, Rui Huang, Shanshan Yin, Zhou Songyang, Junjiu Huang

**Affiliations:** 1 The State Key Laboratory of Ophthalmology, Zhongshan Ophthalmic Center, Sun Yat-sen University, Guangzhou, 510060 China; 2 MOE Key Laboratory of Gene Function and Regulation, Guangzhou Key Laboratory of Healthy Aging Research and State Key Laboratory of Biocontrol, SYSU-BCM Joint Research Center, School of Life Sciences, Sun Yat-sen University, Guangzhou, 510275 China; 3 Department of Dermatology and Skin Science, University of British Columbia, Vancouver, BC V5Z 4E8 Canada; 4 Verna and Marrs Mclean Department of Biochemistry and Molecular Biology, Baylor College of Medicine, One Baylor Plaza, Houston, TX 77030 USA

**Keywords:** lncRNA, Snhg3, mESCs, Self-renewal, Pluripotency

## Abstract

**Background:**

Small nucleolar RNA host gene 3 (Snhg3) is a long non-coding RNA (lncRNA) that was shown to participate in the tumorigenesis of certain cancers. However, little is known about its role in embryonic stem cells (ESCs).

**Methods:**

Here, we investigated the role of Snhg3 in mouse ESCs (mESCs) through both loss-of-function (knockdown) and gain-of-function (overexpression) approaches. Alkaline phosphatase staining, secondary colony formation, propidium iodide staining, western blotting, and quantitative reverse transcription polymerase chain reaction (qRT-PCR) were used to access self-renewal capacity, whereas immunofluorescence, qRT-PCR, and embryoid body formation were performed to examine pluripotency. In addition, the effect of Snhg3 on mouse embryonic development was determined based on the morphological changes, blastocyst rate, and altered pluripotency marker (Nanog, Oct4) expression. Moreover, the relationship between Snhg3 and key pluripotency factors was evaluated by chromatin immunoprecipitation qPCR, qRT-PCR, subcellular fractionation, and RNA immunoprecipitation. Finally, RNA pull-down and mass spectrometry were applied to explore the potential interacting proteins of Snhg3 in mESCs.

**Results:**

We demonstrated that Snhg3 is essential for self-renewal and pluripotency maintenance in mESCs. In addition, Snhg3 knockdown disrupted mouse early embryo development. Mechanistically, Snhg3 formed a positive feedback network with Nanog and Oct4, and 126 Snhg3-interacting proteins were identified in mESCs.

**Conclusions:**

Snhg3 is essential for mESC self-renewal and pluripotency, as well as mouse early embryo development.

**Electronic supplementary material:**

The online version of this article (10.1186/s13287-019-1270-5) contains supplementary material, which is available to authorized users.

## Background

Embryonic stem cells (ESCs) are capable of unlimited propagation (self-renewal) and give rise to cells of all three germ layers (pluripotency), thereby serving as an ideal model to understand the embryonic development, in addition to providing potential cell sources for tissue regeneration [1]. Apart from the intensively studied transcriptional and epigenetic networks that modulate ESC properties [2], a growing body of evidence suggests that long non-coding RNAs (lncRNAs) are also involved in the determination of ESC fate [3–5]. LncRNAs are long transcripts (> 200 nt) that are transcribed by RNA polymerase II, 5′-capped, spliced, and polyadenylated, just like coding mRNAs, but lack protein-coding potential. Thousands of lncRNAs have been identified in human and mouse genomes [6, 7], but only a few are functionally well-characterized with respect to their role in pluripotent stem cells and embryo development [5, 8–10].

Small nucleolar RNA host gene 3 (Snhg3) is a newly discovered lncRNA that was identified as a biomarker of malignant status or poor prognosis in several types of cancers including lung cancer, hepatocellular carcinoma, glioma, ovarian cancer, and colorectal cancer [11–15]. In a previous shRNA-based screen targeting 226 lncRNAs using mouse ESCs (mESCs) [4], Snhg3 was among the 26 lncRNAs for which depletion resulted in decreased luciferase reporter activity from the *Nanog* promoter. However, the detailed functions and associated mechanisms of Snhg3 in mESCs are not clear.

Here, we assessed the expression of Snhg3 in mESCs and during differentiation. Furthermore, we characterized its regulatory functions and associated molecular mechanisms with respect to mESC self-renewal and pluripotency and early mouse embryonic development. Thus, our study not only identify Snhg3 as an additional important player in ESC regulation, but also indicate its potential value for optimizing ESC generation, aimed at accelerating stem cell translational medicine.

## Methods

### Cell culture

The AB2.2 mouse ESC line (passage #18; Darwin Core Facility, Baylor College of Medicine) was maintained under feeder-free conditions on tissue culture dishes coated with 0.1% gelatin (Sigma-Aldrich) in Knockout DMEM medium (Gibco) supplemented with 15% (*v*/*v*) fetal bovine serum, β-mercaptoethanol (55 μM), GlutaMAX-I supplement (2 mM), MEM non-essential amino acids (0.1 mM), LIF (1000 U/ml, Millipore), and 2 inhibitors (CHIR99021, PD325901). To induce differentiation, mESCs were cultured without LIF or with 5 μM retinoic acid (RA) and LIF or subjected to embryoid body (EB) formation as previously described [16].

Mouse embryonic fibroblasts (MEFs) (passage #1; Cyagen, MUIEF-01001) were maintained in DMEM (Corning) with 10% (*v*/*v*) fetal bovine serum (Gibco) and 1% penicillin-streptomycin (Gibco).

### siRNA transfection

mESCs were transfected with siRNA oligos targeting indicated genes for 48 h using Lipofectamine 2000 (Invitrogen) according to the manufacturer’s instructions. The siRNA oligos were purchased from Genepharma, and their sequences are listed in Additional file 2: Table S1.

### Quantitative real-time PCR

Total RNA was isolated using the RNeasy Mini Kit (Qiagen). After cDNA synthesis with the iScript Select cDNA Synthesis Kit (BioRad), qRT-PCR was performed using SYBR Green PCR Master Mix (Applied Biosystems) with an ABI StepOnePlus Real-Time PCR System. The results were normalized to *GAPDH* transcripts and analyzed using the delta-delta Ct method to calculate the relative fold change in gene expression. Primer sequences are listed in Additional file 2: Table S2.

### Alkaline phosphatase staining

AP staining was performed using the alkaline phosphatase detection kit (SCR004, Millipore) or the Vector blue alkaline phosphatase substrate kit (SK-5300; Vector Laboratories) following the manufacturers’ instructions.

### Secondary colony formation

Before the assay, MEFs were treated with 10 μg/ml of Mitomycin C (Sigma) for 3 h to serve as feeders. mESCs were then re-plated at different densities (200, 400, or 800 cells/well) onto feeders in 6-well culture dishes to form secondary ES cell colonies for 7 days. AP staining was performed at day 7.

### Western blotting and immunofluorescence staining

For western blotting (WB), cells were harvested and lysed with RIPA buffer at 90 °C. Proteins were separated by SDS-PAGE and transferred to polyvinylidene fluoride membranes (BioRad, 1620177) for blotting with appropriate antibodies. For immunofluorescence staining (IF), cells grown on glass cover slips were fixed with 4% paraformaldehyde, permeabilized with 0.2% Triton X-100, treated with 2% BSA, and probed with indicated antibodies. Images were captured using a Zeiss inverted microscope.

The following antibodies were used for WB: anti-caspase3 (#9662; Cell Signaling Technology), anti-cleaved caspase3 (#9661; Cell Signaling Technology), and anti-GAPDH (sc-25778; Santa Cruz). For immunostaining, the antibodies included anti-Oct4 (sc-5279; Santa Cruz) and anti-Nanog (Ab80892; Abcam). DAPI (Sigma) was used to stain the nuclei.

### Flow cytometry analysis

For cell apoptosis analysis, cells were stained with an Annexin V-propidium iodide (PI) apoptosis detection kit (BD Bioscience) according to the manufacturer’s instructions. As for cell cycle analysis, cells were harvested, washed, and fixed in 70% ethanol overnight at 4 °C. The next day, cells were centrifuged, washed, and incubated with PI for 30 min. Cell apoptosis rate or cell cycle phase analysis was performed using a FACScalibur flow cytometer (BD Bioscience).

### Cell proliferation analysis

Cell proliferation was measured via CCK-8 assay (Dojindo) according to the manufacturers’ instructions. Proliferation rates were determined at 0, 24, 48, and 72 h.

### Stable cell line generation

To construct Snhg3-overexpressing mESCs, HEK293T cells were transfected with pLenti-HA-Flag vector expressing mouse full-length *Snhg3*, psPAX2, and PMD2.G using Lipofectamine 2000 (Invitrogen). Virus was collected 48 h and 72 h after transfection and used to transduce mESCs in the presence of Polybrene (8 μg/ml). Cells were selected with puromycin (2 μg/ml) for 1 week. GFP-overexpressing mESCs were generated as a control. The PCR primers for Snhg3 cloning were as follows: FP, 5′-GACTTCCGGGCGTTACTTAA-3′; RP, 5′-AGACATTCAAATGCTTTAAT-3′.

To construct Snhg3-knockdown mESCs, cells were transfected with a pLKO.pig plasmid expressing shRNA against Snhg3, and stable cells were selected with puromycin for 1 week [17]. shRNA-targeting luciferase was constructed as a control. The target sequences were as follows: sh*Snhg3*-1, CACCTACTGAATAGTTATTAT; sh*Snhg3*-2, TCAATGATTTCAGGTACTTTG; and sh*Control*, CTTACGCTGAGTACTTCGA.

### Zygote collection, knockdown treatment, culture, and analysis

The collection of zygotes was performed as previously described [18]. CD1 female mice were super-ovulated with 5 IU PMSG (367222, Calbiochem) and 5 IU hCG (230734, Calbiochem) for 46 h and used for breeding. After release from the oviduct ampullae, the zygotes were injected with siRNA oligos (20 μM) using the Femojet microinjection system (Eppendorf) and then cultured in KSOM medium for different amounts of time.

After culturing the zygotes for 24 h (two-cell stage), whole transcriptome amplification was performed using the PEPLI-g WTA single cell kit (Qiagen, 150063). Following cDNA synthesis and amplification, the product was used for qRT-PCR to access the knockdown efficiency and the expression of pluripotency markers.

For whole-mount staining, blastocysts were collected into embryo GPS dishes (LifeGlobal Group), fixed with 4% paraformaldehyde, permeabilized in 0.1% Triton X-100, blocked with 3% BSA solution, and blotted with anti-Oct4 (sc-5279; Santa Cruz) and anti-Nanog (Ab80892; Abcam) antibodies.

### Cellular fractionation

Cytoplasmic and nuclear RNA were isolated and purified using the Cytoplasmic and Nuclear RNA Purification Kit (Norgen, Canada) according to the manufacturer’s instructions. The expression levels of *Snhg3*, *Gapdh*, and *Xist* were detected by qRT-PCR.

### Chromatin immunoprecipitation

Chromatin immunoprecipitation (ChIP) assays were performed using the Magna ChIP Kit (Millipore) according to the manufacturer’s instructions. Briefly, the crosslinked chromatin was sonicated into 200–300-bp fragments, and the lysates were immunoprecipitated with antibodies against Nanog (BL1663, Bethyl), Oct4 (ab181557, Abcam), or Sox2 (ab97959, Abcam), or with control IgG (ab37415, Abcam). The precipitated chromatin DNA was recovered and assessed by qRT-PCR.

### RNA immunoprecipitation assay

For this, 10^7^ cells were harvested; resuspended in 2 ml PBS, 2 ml nuclear isolation buffer (1.28 M sucrose, 40 mM Tris-HCl pH 7.5, 20 mM MgCl_2_, 4% Triton X-100), and 6 ml of water; and then incubated on ice for 20 min. The nuclei were pelleted by centrifugation at 2500×*g* for 15 min and resuspended in 1 ml of RNA immunoprecipitation (RIP) buffer (150 mM KCl, 25 mM Tris pH 7.4, 5 mM EDTA, 0.5 mM DTT, 0.5% NP40, 100 U/ml RNAase inhibitor, 1 mM PMSF, and protease inhibitors). Chromatin was sheared using a Dounce homogenizer for 15 strokes and centrifuged at 15,000×*g* for 10 min. Next, 2 μg of antibody and corresponding IgG was added to the lysate and incubated overnight at 4 °C with rotation. The next day, protein A/G beads (40 μl) were added for 1 h at 4 °C. The beads were pelleted at 600×*g* for 30 s and resuspended in 500 ml of RIP buffer. Washes were repeated six times. The beads were resuspended in 1 ml Trizol reagent, and the manufacturer’s instructions were followed to purify RNA. Finally, the purified RNA was subjected to qRT-PCR analysis.

### RNA pull-down assay and mass spectrometry

RNA pull-down assays were performed as described [5]. Briefly, mouse full-length *Snhg3* was cloned into the pcDNA3.1(+) vector, transcribed in vitro, biotin-labeled using the Biotin RNA labeling Mix (Roche) and MEGA shortscript T7 kit (Ambion), and purified with an RNeasy Mini Kit (Qiagen). Then, 3 μg of biotinylated RNA was incubated at 90 °C for 2 min, placed on ice for 2 min, and then shifted to room temperature for 20 min for proper structure formation. Folded RNA was mixed with 1 mg of mESC cell lysates in RIP buffer and incubated at 4 °C for 1 h. Next, 20 μl of Streptavidin Agarose Beads (Invitrogen) were added to the mixture and incubated at 4 °C for 2 h. The beads were washed briefly in RIP buffer five times, and the complexes were eluted, resolved by SDS-PAGE gel, and stained with Silver Stain Plus (Bio-Rad). Gels were sent to Beijing Proteome Research Center for mass spectrometry analysis. Gene classification of biological processes, protein classes, and molecular functions was performed based on the Panther database and visualized with ggplot2 package in R environment. The proteins identified from mass spectrometry are listed in Additional file 3: Table S3.

### Published data analysis

ChIP sequencing data (GSE53490, GSE73952) were retrieved from the NCBI GEO database.

### Statistical analysis

The data were reported as mean ± standard deviation (SD). Significant differences between the groups were calculated by performing a chi-square test or a two-way ANOVA and were defined as *p < 0.05, **p < 0.01, and ***p < 0.001.

## Results

### Snhg3 is highly expressed in mESCs and decreased after differentiation

**Fig. 1:**
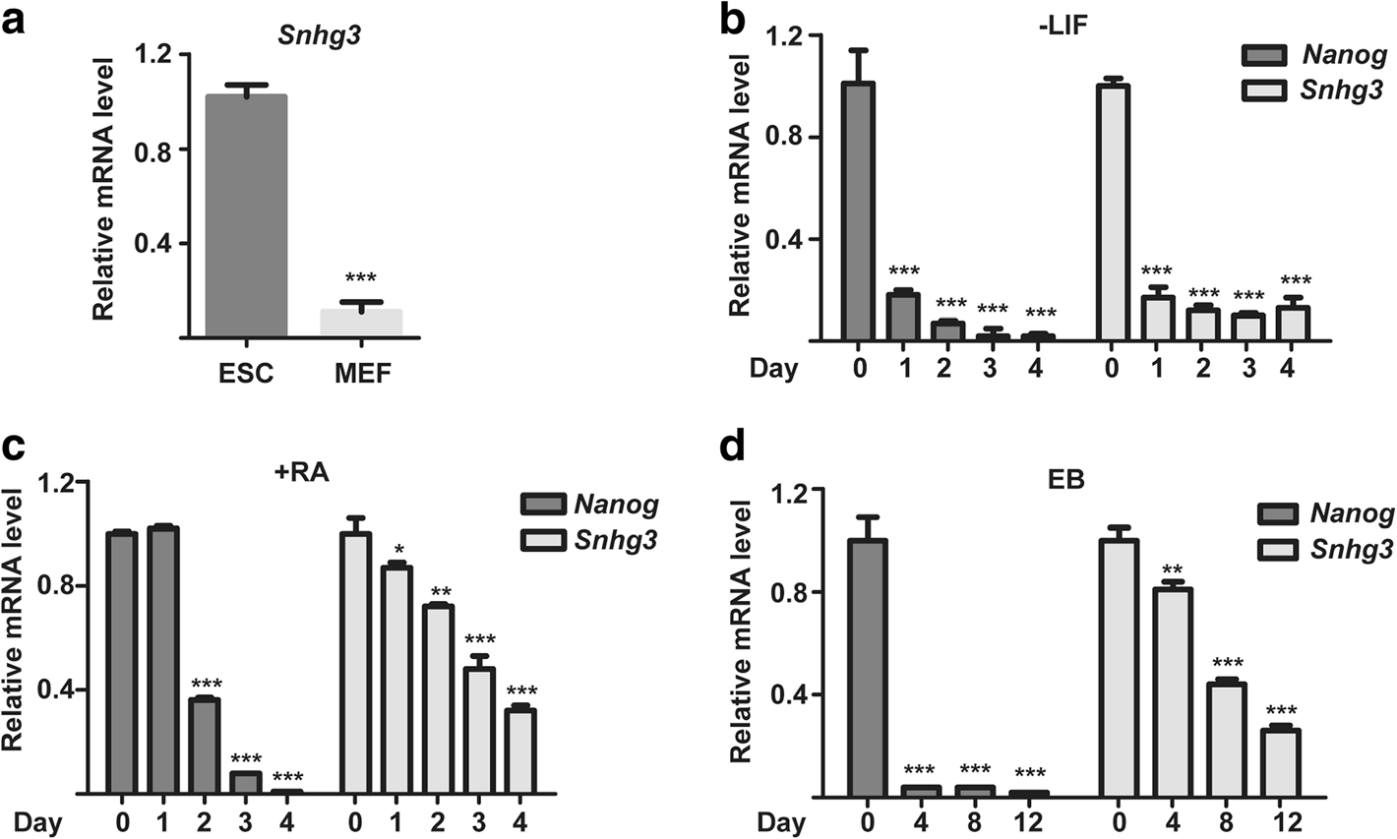
Snhg3 is enriched in mouse embryonic stem cells (mESCs). **a** qRT-PCR analysis of *Snhg3* expression in mESCs and mouse embryonic fibroblasts (MEFs). To induce differentiation, mESCs were cultured without LIF (**b**), with 5 μM retinoic acid (RA) (**c**), or as embryoid bodies (EBs) (**d**) for the indicated amounts of time. Cells or EBs were collected for qRT-PCR to detect *Snhg3* expression. *Nanog* was used as a positive control. Data are presented as mean ± SD; *n* = 3, two-way ANOVA. *p < 0.05, **p < 0.01, ***p < 0.001 for all panels

To explore the potential functions of Snhg3 in mESCs, we first compared the expression levels between mESCs and MEFs. qRT-PCR analysis showed that the expression of Snhg3 was much higher in mESCs than in MEFs (~ 9-fold; Fig. 1a). To further investigate the expression patterns of Snhg3 during differentiation, we adopted three different methods to induce mESC differentiation, including LIF withdrawal, RA addition, and EB formation. Similar to levels of the key pluripotency marker *Nanog*, the expression of Snhg3 was gradually downregulated after differentiation (Fig. 1b). Thus, the enrichment of Snhg3 in an undifferentiated state suggested its potential regulatory function in mESCs.

### Knockdown of Snhg3 impairs mESC self-renewal

**Fig. 2:**
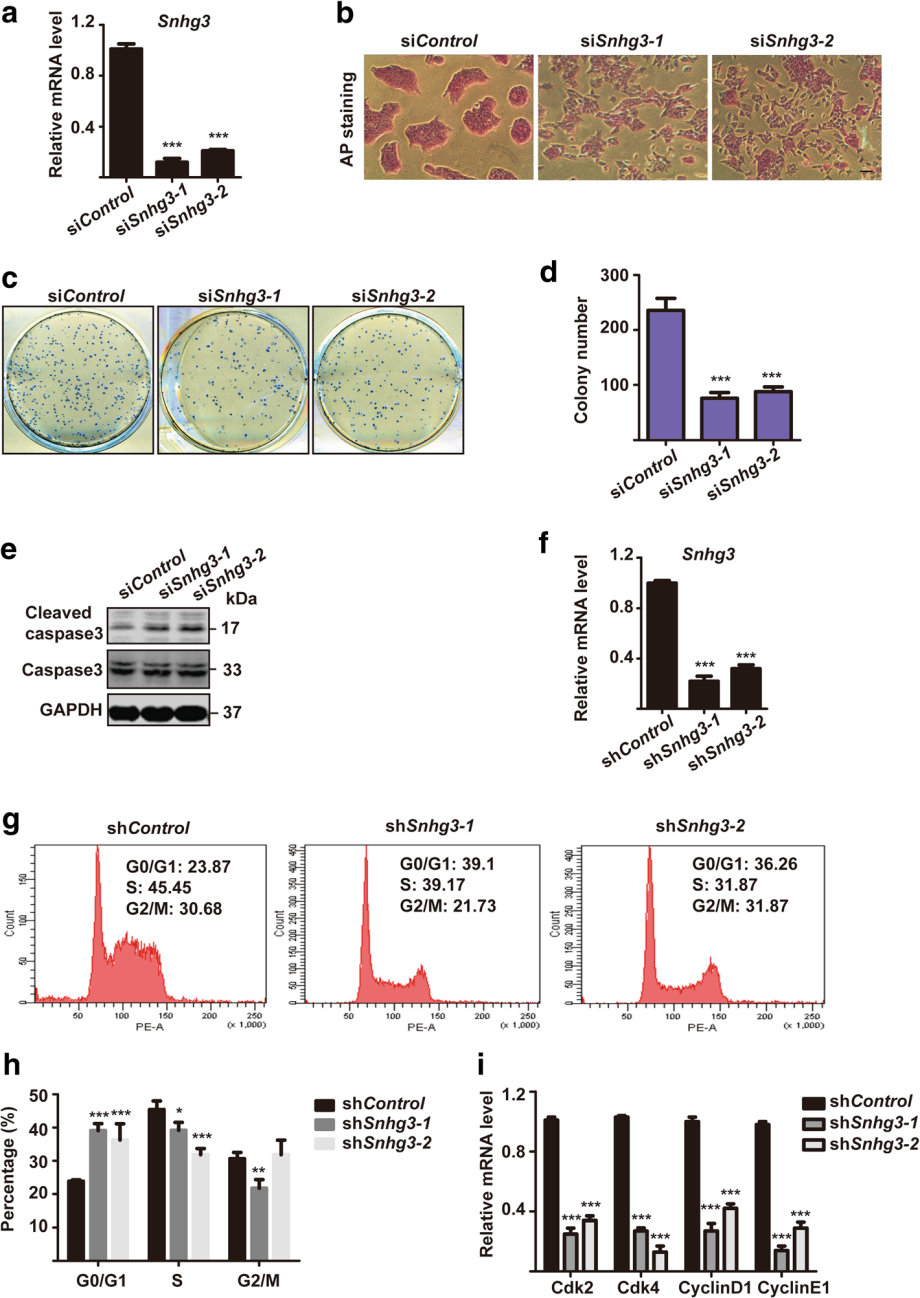
Snhg3 depletion impairs mouse embryonic stem cell (mESC) self-renewal. **a** mESCs were transfected with si*Control* or two different oligos targeting *Snhg3* for 48 h and subjected to qRT-PCR to access knockdown efficiency. **b** Alkaline phosphatase (AP) staining of mESCs after si*Control* or si*Snhg3* treatment; × 4 objective, scale bar = 100 μm. si*Control*- or si*Snhg3*-treated mESCs were re-plated onto feeders to form secondary ESC colonies for 7 days, which was followed by AP staining (**c**). The numbers of total ESC colonies were quantified in **d**. **e** si*Control*- or si*Snhg3*-treated mESCs were lysed for western blotting with indicated antibodies. GAPDH was used as a loading control. **f** Two stable *Snhg3*-knockdown mESCs were generated using a lentivirus. Knockdown efficiency was measured by qRT-PCR. **g** Cell cycle distribution of sh*Control* or sh*Snhg3* cell lines was determined by flow cytometry. **h** Quantification of the percentage of cells in different cell cycle phases. **i** qRT-PCR analysis of cell cycle-related markers in sh*Control* or sh*Snhg3* cell lines. Data are presented as mean ± SD; *n* = 3, two-way ANOVA. *p < 0.05, **p < 0.01, ***p < 0.001 for all panels

To understand the role of Snhg3 in mESCs, we first transfected two efficient and specific targeting Snhg3 siRNA oligos into mESCs to knock down its expression (Fig. 2a). After transfection for 48 h, si*Snhg3*-treated mESCs quickly lost their mESC morphology, became flat, and displayed weaker AP activity, when compared to those features in control cells (Fig. 2b). To further access whether Snhg3 regulates self-renewal in mESCs, we performed secondary colony formation assays by re-plating 800 si*Control* or si*Snhg3*-treated mESCs on feeders for 7 days (Fig. 2c). As indicated by AP staining, depletion of Snhg3 significantly impaired the colony formation ability of mESCs (Fig. 2d). We hypothesized that this might be a consequence of apoptosis triggered by Snhg3 depletion. To test this hypothesis, we examined the expression of cleaved caspase 3, a marker of apoptosis, by western blotting. Indeed, cleaved caspase 3 levels were increased upon Snhg3 knockdown, whereas total caspase 3 remained stable (Fig. 2e). In addition, cell apoptosis was also analyzed via Annexin V and PI staining. Flow cytometry results showed that Snhg3 knockdown led to more early apoptotic cells, and less viable cells, which suggested that Snhg3 depletion results in apoptosis (Additional file 1: Figure S1a-b).

To understand the mechanism associated with suppressed self-renewal observed in *Snhg3*-knockdown mESCs, we first generated stable *Snhg3*-knockdown mESCs (sh*Snhg3*-1 and sh*Snhg3*-2) using a lentivirus delivery system; shRNA targeting luciferase served as a negative control (sh*Control*) (Fig. 2f). The cell cycle profile of sh*Snhg3* mESCs was analyzed by PI staining, followed by flow cytometry (Fig. 2g). Notably, sh*Snhg3* mESCs showed a marked increase in the proportion of cells in G0/G1 phase, and a concomitant decreases in S phase populations, as compared to those in the sh*Control* cells, indicating that *Snhg3* depletion leads to cell cycle arrest at the G1 phase (Fig. 2h). Consistently, the expression of genes encoding cell cycle-related molecules including *Cdk2*, *Cdk4*, *CyclinD1*, and *CyclinE1* was reduced in *shSnhg3* mESCs (Fig. 2i). These data indicated that the disruption of mESC self-renewal by Snhg3 depletion was partially mediated by cell cycle perturbation and apoptosis induction.

### Snhg3 depletion alters mESC pluripotency

**Fig. 3:**
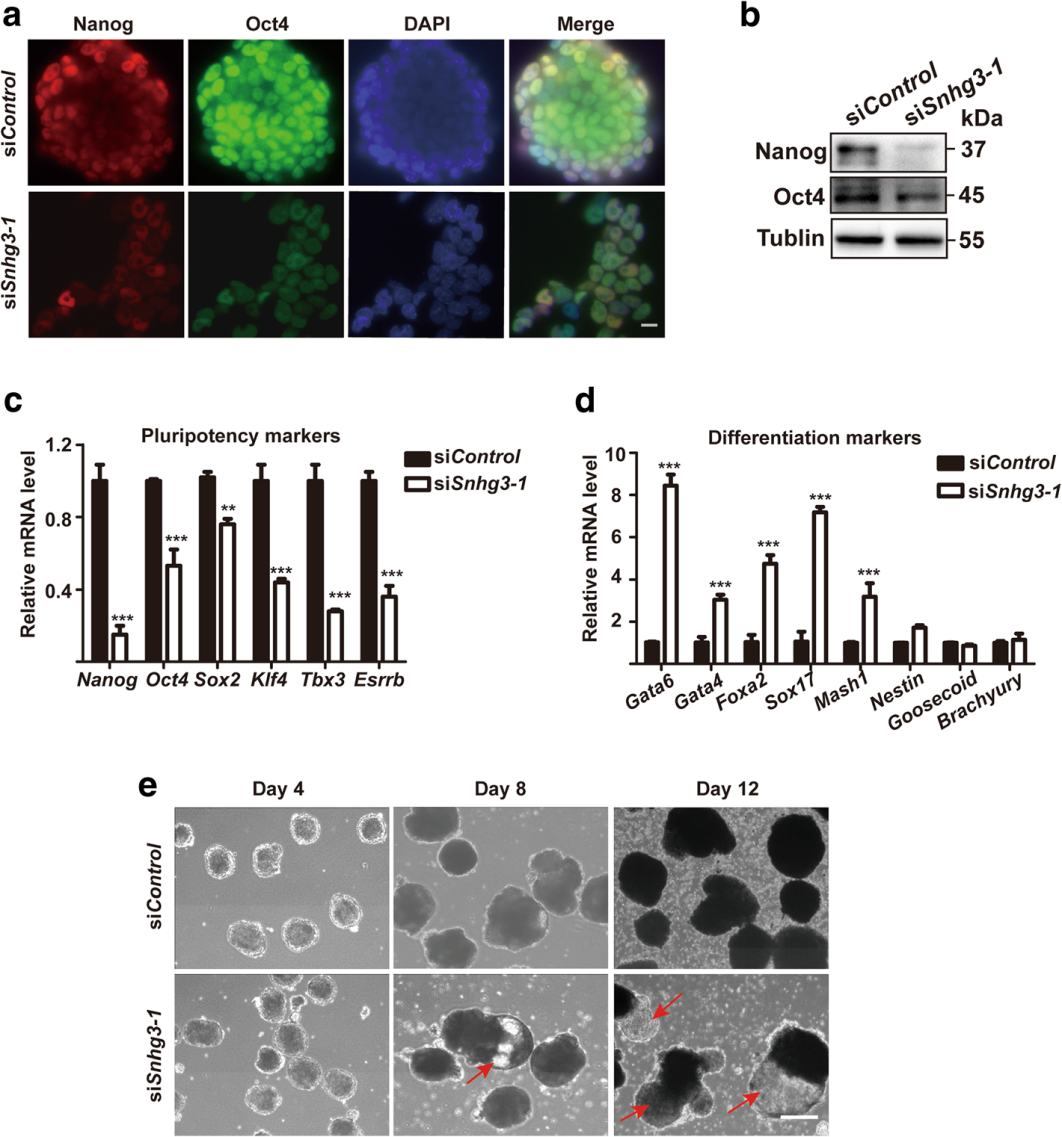
Snhg3 depletion resulted in mouse embryonic stem cell (mESC) differentiation. Immunostaining (**a**) and western blot (**b**) of mESCs after si*Control* treatment or si*Snhg3* depletion to detect Nanog and Oct4 expression. The nuclei were stained with DAPI; × 20 objective, scale bar = 100 μm. Tublin was used as a loading control. qRT-PCR was used to analyze the expression levels of pluripotency markers (**c**) or differentiation markers (**d**) after si*Control* treatment or si*Snhg3* knockdown in mESCs. Data are presented as mean ± SD; *n* = 3, two-way ANOVA. **p < 0.01; ***p < 0.001. **e** si*Control*- or si*Snhg3*-treated mESCs were cultured as hanging drops in ES medium without LIF to form embryoid bodies (EBs) for the indicated times. Red arrows indicate cystic EB formation; × 4 objective, scale bar = 400 μm

To determine whether Snhg3 also affects the pluripotency in mESCs, we performed immunostaining (IF) and western blot to probe for the expression levels of the key pluripotency factors Nanog and Oct4 after knocking down Snhg3 in mESCs; both markers were dramatically reduced compared to the expression in si*Control* cells (Fig. 3a, b). qRT-PCR also confirmed that Snhg3 knockdown repressed the expression of several pluripotency markers including *Nanog*, *Oct4*, *Sox2*, *Klf4*, *Tbx3*, and *Esrrb* (Fig. 3c). We then speculated that Snhg3 depletion might lead to mESC differentiation. Therefore, we further examined the expression patterns of three germ layer markers in these cells by qRT-PCR. As shown in Fig. 3d, si*Snhg3*-treated mESCs were primed for differentiation, which was characterized by elevated expression levels of endoderm markers (*Gata6*, *Gata4*, *Foxa2*, *Sox17*) and the ectoderm marker *Mash1*, although Snhg3 knockdown had only a marginal effect on expression of the ectoderm gene *Nestin* and mesoderm genes (*Goosecoid*, *Brachyury*) (Fig. 3d). Finally, to validate the differentiation state of *Snhg3*-knockdown mESCs, we performed an EB formation assay for 12 days to mimic early mouse embryonic development [19]. Importantly, cystic EBs, which contain primitive endoderm-derived cells, appeared early at day 8 after *Snhg3* depletion and became larger by day 12 (Fig. 3e). In contrast, few cystic EBs appeared in the si*Control* group even after 12 days (Fig. 3e). These results demonstrated that Snhg3 is required for maintaining the pluripotency of mESCs.

### Snhg3 overexpression promotes self-renewal and represses mESC differentiation

**Fig. 4:**
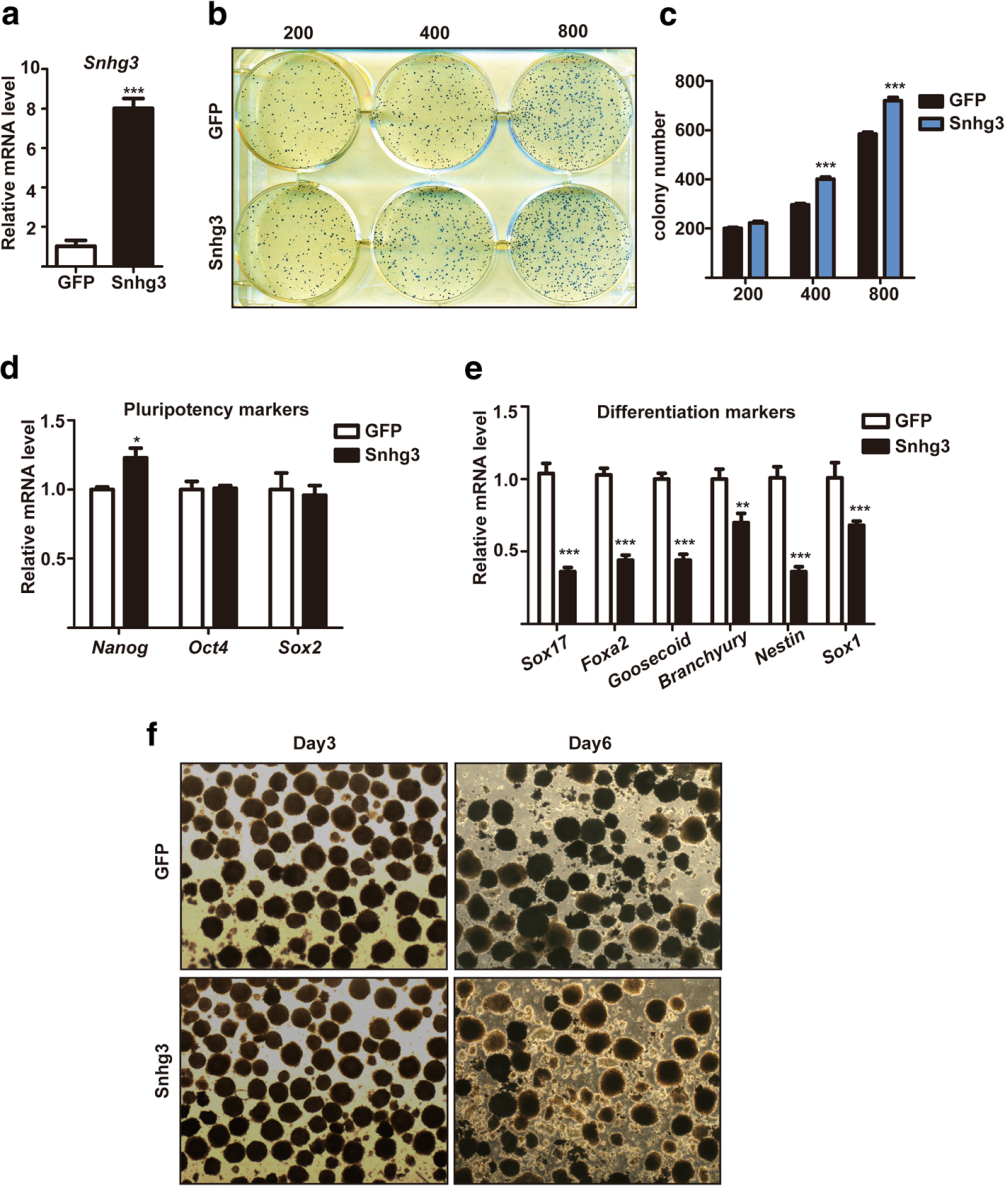
Snhg3 overexpression promotes mouse embryonic stem cell (mESC) self-renewal and inhibits differentiation. **a** Stable Snhg3-overexpressing mESCs were generated using a lentivirus. GFP-mESCs were generated as a control. qRT-PCR was used to access *Snhg3* levels. Secondary colony formation assays were performed by re-plating different cell numbers (200, 400, or 800 cells per well) onto feeders for 7 days (**b**). Total colony numbers were quantified after alkaline phosphatase (AP) staining (**c**). qRT-PCR analysis of the expression of pluripotency markers (**d**) or differentiation markers (**e**) in Snhg3- or GFP-overexpressing mESCs. **f** Embryoid bodies (EBs) formed by GFP- or Snhg3-overexpressing mESCs were visualized by phase contrast microscopy after day 3 or 6; × 4 objective. Data are presented as the mean ± SD; *n* = 3, two-way ANOVA. *p < 0.05, **p < 0.01, ***p < 0.001 for all panels

Next, we generated stable Snhg3-overexpressing mESCs using a lentivirus delivery system, in which GFP-overexpressing mESCs served as a negative control. After validating the constitutive overexpression of *Snhg3* transcripts (Fig. 4a), we examined the self-renewal of these cells by performing secondary colony formation assays, in which the cells were plated at different densities on feeders for 7 days. Compared to that with GFP-overexpressing mESCs, more AP-stained colonies were observed after plating 400 or 800 Snhg3-overexpressing mESCs (Fig. 4b, c), indicating robust cell proliferation after Snhg3 overexpression. Consistently, increased cell growth after Snhg3 overexpression was also observed via CCK-8 assay (Additional file 1: Figure S1c). Additionally, only a slight increase in the expression of *Nanog* was observed, although the other two pluripotency factors, namely *Oct4* and *Sox2*, were not affected (Fig. 4d). Notably, Snhg3 overexpression significantly inhibited expression of markers of the three germ layer, as confirmed by qRT-PCR (Fig. 4e), suggesting that Snhg3 expression is sufficient to perturb differentiation. Moreover, after EB formation for 6 days, Snhg3-overexpressing EBs became smaller, dispersed, and irregular when compared to control EBs, indicating that the differentiation process was dysregulated (Fig. 4f). Thus, these data demonstrated that ectopic Snhg3 expression promotes self-renewal and blocks differentiation in mESCs.

### Snhg3 is required for early mouse embryonic development

**Fig. 5:**
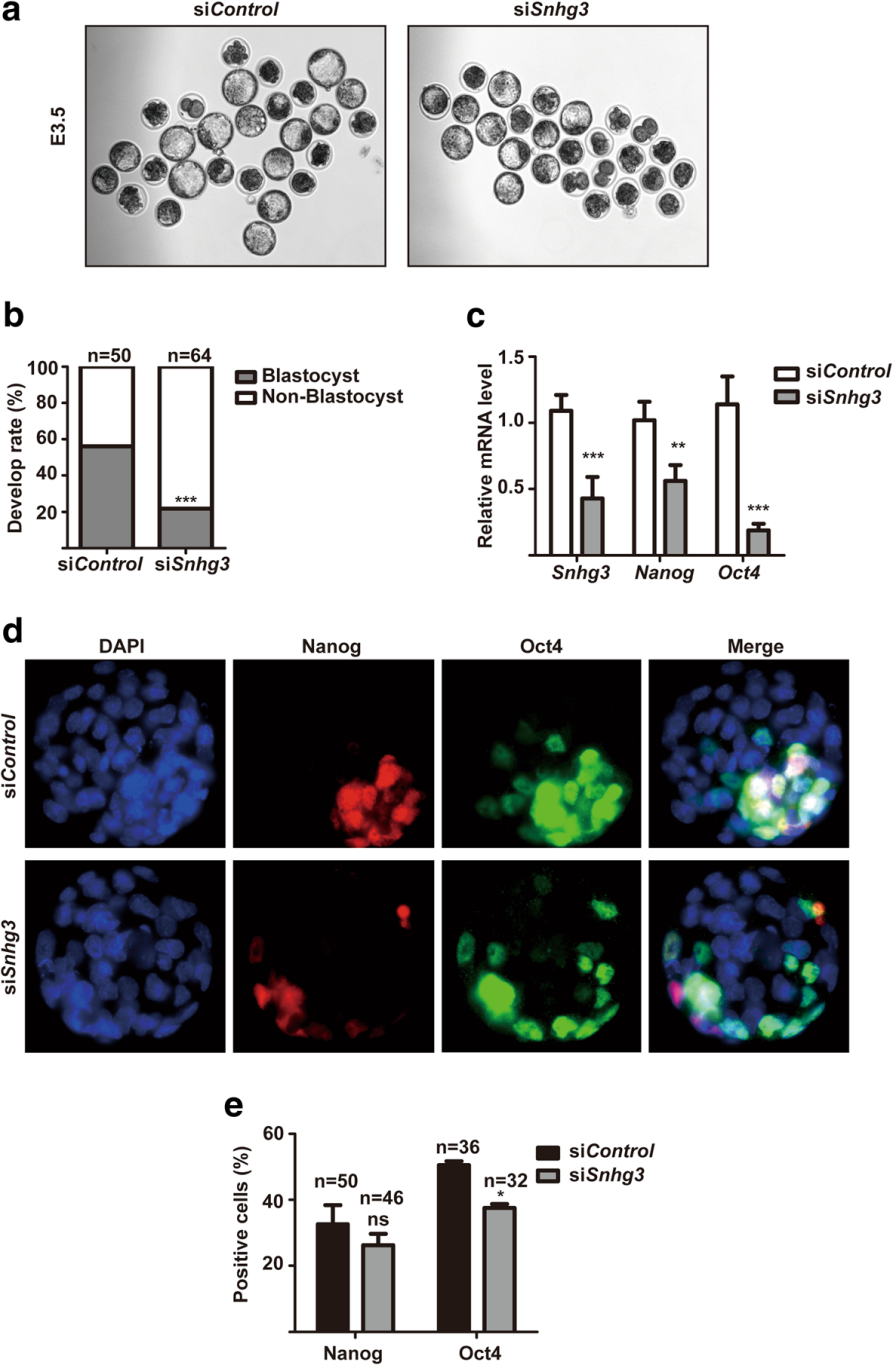
Snhg3 is essential for mouse embryonic development. **a** Morphology of E3.5 mouse embryos after si*Control* or si*Snhg3* treatment; × 10 objective. **b** si*Control*- or si*Snhg3*-injected zygotes were cultured in vitro until E4.5 and the blastocyst/non-blastocyst rate was quantified. si*Control*, *n* = 50; si*Snhg3*, *n* = 64. ***p < 0.001, chi-square test. **c** qRT-PCR analysis of *Snhg3*, *Nanog*, and *Oct4* expression in blastocysts after si*Control* or si*Snhg3* treatment. Data are presented as the mean ± SD; *n* = 3, two-way ANOVA. **p < 0.01, ***p < 0.001. **d** Immunostaining of Nanog (green) and Oct4 (red) in blastocysts after si*Control* or si*Snhg3* treatment. The nuclei were stained with DAPI (blue); × 20 objective. **e** The percentage of Nanog- or Oct4-positive cells in blastocysts was quantified based on the staining in **d**. *n*, number of embryos analyzed. *p < 0.05, two-way ANOVA

Currently, the role of Snhg3 in mouse embryo development was unknown. To assess its potential function, zygotes were microinjected with siRNAs against *Snhg3* or *Control* oligonucleotides, and the morphology and development rate were observed and recorded at E3.5. We found that whereas 56% of control embryos developed normally to the blastocyst stage (*n* = 50), the blastocyst development rate for *Snhg3*-depleted embryos dropped to 21% (*n* = 64) (Fig. 5a, b). Moreover, qRT-PCR results indicated that after *Snhg3* knockdown, the mRNA levels of *Nanog* and *Oct4* were decreased to around 50% and 20% of control levels, respectively (Fig. 5c). Similarly, IF staining also showed that the number of Oct4-positive cells was decreased in *Snhg3*-depleted embryos, whereas the Nanog-positive cell number was only slightly changed (Fig. 5d, e). These data suggested that Snhg3 plays an important role during early embryonic development.

### Snhg3 functions with Nanog and Oct4 in mESCs

**Fig. 6:**
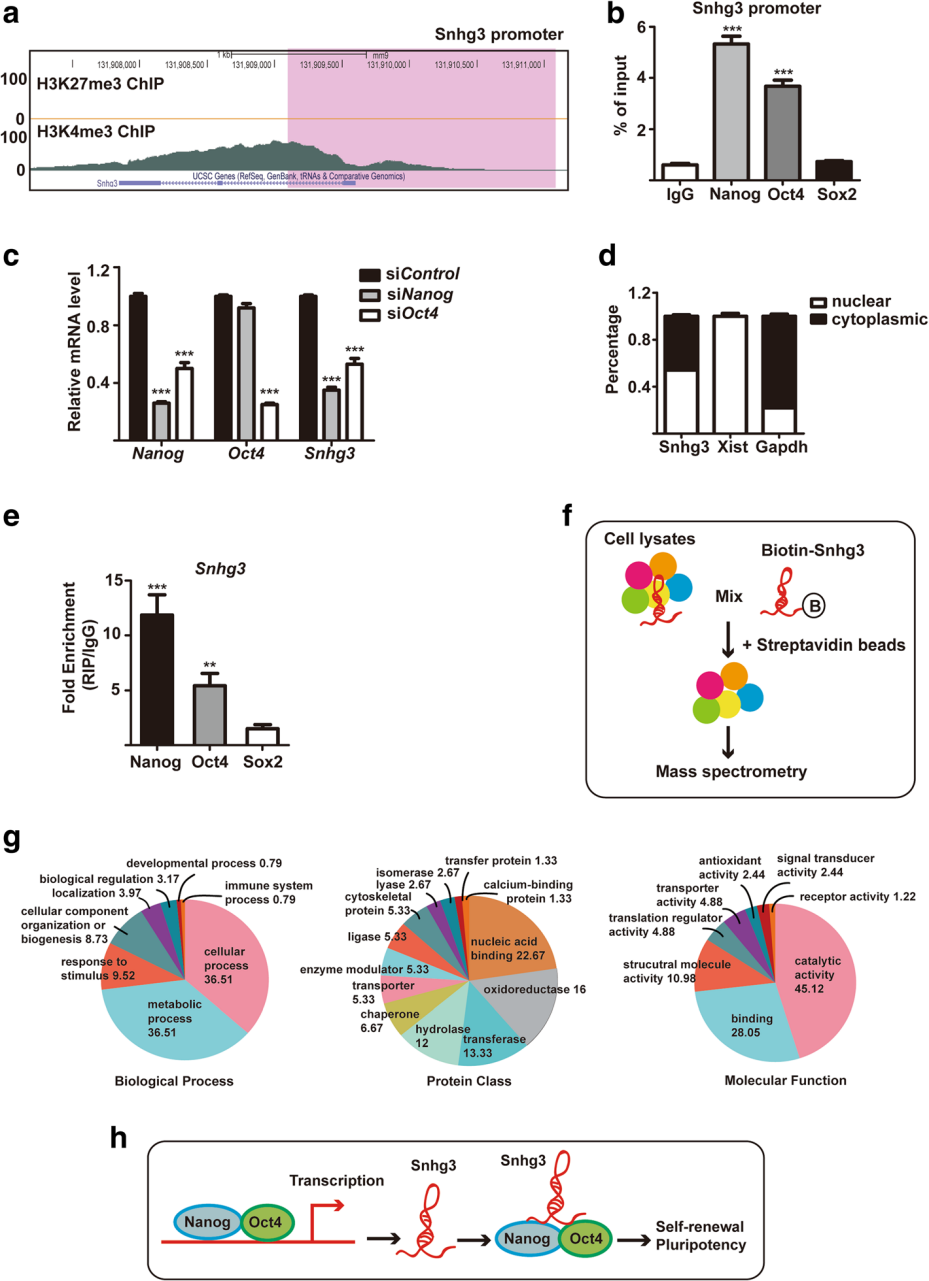
Snhg3 is regulated by pluripotency factors and its associated protein network. **a** Genome browser plot of histone methylation related to transcriptional activation (H3K4me3) and repression (H3K27me3) in the gene body region of *Snhg3* in mouse embryonic stem cells (mESCs). The gray peaks indicate potential H3K4me3-binding sites in Snhg3. The purple box highlights the proximal promoter regions (− 0.5 kb to + 1.5 kb of transcriptional start site) of *Snhg3*. **b** ChIP-qPCR analysis of Nanog, Oct4, or Sox2 occupancy in the *Snhg3* promoter region in mESCs. IgG was used as a negative control. **c** qRT-PCR analysis of the expression levels of *Nanog*, *Oct4*, and *Snhg3* after knocking down *Nanog* or *Oct4* in mESCs. **d** Cellular fractionation was performed in mESCs, and mRNA levels of *Snhg3* were measured by qRT-PCR. *Xist* and *Gapdh* served as nuclear and cytoplasmic markers, respectively. The percentage of subcellular fractions of each gene is shown. **e** RNA immunoprecipitation assay was performed by using anti-Nanog, anti-Oct4, or anti-Sox2 antibodies to precipitate Snhg3 in mESCs. IgG was used as a negative control. **f** Flowchart of RNA pull-down and mass spectrometry (MS) for Snhg3-associated proteins. **g** Pie charts of Gene Ontology (GO) term analysis of the 126 total Snhg3-associated proteins identified by MS. **h** Model for lncRNA *Snhg3* function in mESCs. Snhg3 is bound and activated by pluripotency factors Nanog and Oct4 in mESCs and can form a protein complex with Nanog and Oct4, which in term safeguards self-renewal and pluripotency in mESCs. Data are presented as mean ± SD; *n* = 3, two-way ANOVA. **p < 0.01, ***p < 0.001 for all panels

To understand the role of Snhg3 at the molecular level, we first searched for its upstream regulators in mESCs using published ChIP-seq data (GSE73952, GSE53490) [20, 21]. Notably, we found that the active histone marker tri-methylation of histone 3 lysine 4 (H3K4me3) was enriched at the promoter of *Snhg3*, whereas the repressive histone marker tri-methylation of histone 3 lysine 27 (H3K27me3) was undetectable, indicating that the *Snhg3* transcript was activated in mESCs and that it was in part regulated by an epigenetic mechanism (Fig. 6a). Further, increasing evidence suggests that the transcription of lncRNAs is activated by the core pluripotency transcription factors in ESCs [4, 22]. Therefore, we performed ChIP assays to examine the binding of Nanog, Oct4, and Sox2 to the *Snhg3* promoter. IgG was used as a negative control (Fig 6b). ChIP-qPCR results confirmed the enrichment of Nanog and Oct4, whereas Sox2 was not found, at the *Snhg3* promoter region (Fig. 6b). To further validate the transcriptional activation of Snhg3 by Nanog and Oct4, qRT-PCR analysis was conducted after downregulating *Nanog* or *Oct4* using siRNAs in mESCs. The results showed that *Nanog* or *Oct4* knockdown significantly decreased the expression of *Snhg3* (Fig. 6c). Thus, these results suggested that Nanog and Oct4 are upstream regulators of Snhg3 in mESCs.

To determine how Snhg3 modulates its downstream target genes in mESCs, we first performed subcellular fractionation analysis to delineate the localization of Snhg3. qRT-PCR results demonstrated the *Snhg3* transcripts were distributed similarly in both the cytoplasm and nucleus (Fig. 6d), suggesting that Snhg3 might exert its function at both the transcriptional and post-transcriptional levels. Since we proved that Snhg3 is directly targeted and regulated by Nanog and Oct4, and that the expression of Nanog and Oct4 are also decreased after Snhg3 knockdown (Fig. 3b), we inferred that there might be a feedback loop between Snhg3 and these two pluripotency factors in mESCs. To test this, we performed RIP assays using Nanog and Oct4 antibodies. Sox2 antibody and IgG were used as controls. qRT-PCR showed that there was a substantial enrichment of Snhg3 after the pull-down assays with Nanog and Oct4 antibodies (Fig. 6e). In contrast, no significant enrichment of Snhg3 was found with anti-Sox2 (Fig. 6e). These data indicated that Snhg3 can bind Nanog and Oct4 in mESCs. Finally, to explore potential interacting partners of Snhg3 in mESCs, we performed RNA pull-down assays followed by mass spectrometry and identified 126 Snhg3-bound proteins (Fig. 6f, Additional file 3: Table S3). Bioinformatics analysis based on the Gene Ontology (GO) of identified proteins was then performed using the PANTHER database (Fig. 6g). Under the “Biological Process” classification, large proportions of genes were involved in metabolic and cellular processes. For the “Protein Class,” categories of nucleic acid binding and oxidoreductase were significantly enriched, whereas for the “Molecular Function,” catalytic activity and binding were significantly enriched (Fig. 6g). Thus, these data suggested the possible involvement of Snhg3 in metabolic regulation or as a scaffold to recruit proteins or ncRNAs in mESCs.

## Discussion

Accumulating data have shown that lncRNAs, which were previously considered as transcriptional noise, participate in multiple biological processes [23]. Although lncRNAs are expressed at a lower level than protein-coding genes, they display more tissue- or cell type-specific expression patterns [24]. Therefore, lncRNAs have been considered essential regulators of ES cell maintenance or differentiation. For example, lnc*Kdm2b* maintains mESC self-renewal via transcriptional activation of the pluripotency factor Zbtb3 [8]. In contrast, linc*ROR* functions as a competing endogenous RNA (ceRNA) for miR-145, which targets mRNAs encoding core pluripotency genes, thereby promoting the self-renewal in hESCs [25]. Furthermore, Lnc*PRESS1* augments pluripotency in hESCs by impairing SIRT6-mediated histone H3K56 deacetylation at the promoter regions of pluripotency genes [9]. Thus, these studies indicate that lncRNAs can adopt transcriptional, post-transcriptional, and epigenetic regulatory roles to modulate gene expression in ESCs.

Snhg3 is a lncRNA that has been mainly found to be associated with tumorigenesis. Previously, the function of Snhg3 in ESCs was only described in an RNAi screen study, in which its depletion impaired mESC morphology and downregulated the expression of several pluripotency factors including Nanog, Oct4, Sox2, Klf4, and Zfp42 [4]. However, how Snhg3 precisely exerts its effect on mESCs was not characterized. Here, we used both loss-of-function and gain-of-function strategies to demonstrate that Snhg3 can promote the self-renewal and pluripotency of mESCs, which mainly occurs through associations with the core pluripotency factors Nanog and Oct4 via potential feedback loops (Fig. 6h). In addition, our data also implied an important role for Snhg3 in early mouse embryonic development.

Mouse Snhg3 is located on chromosome 4 (chr4: 132,351,934-132,353,633, UCSC Genome Browser/mm10) and is transcribed on the reverse strand. Interestingly, we found that knocking down *Snhg3* in mESCs also reduced the mRNA level of its neighbor gene Rcc1 (data not shown), a guanine-nucleotide exchange factor for Ran GTPase and plays roles in mitosis, nuclear envelope assembly. The role of Rcc1 in ESCs is not characterized before, yet a paper has found it may be a pluripotency marker in embryonal carcinoma cell line [26]. Thus, besides interacting with pluripotency factors, Snhg3 may also work with Rcc1 to regulate stemness in ESCs.

Snhg3 was distributed at a similar ratio in both the nucleus and cytoplasm. We have studied the functions of nuclear Snhg3; however, the role of cytoplasmic Snhg3 needs to be explored. It is possible that Snhg3 can regulate the alternative splicing of pre-mRNA transcripts, modulate mRNA transportation, or act as a ceRNA for miRNAs.

LncRNAs also tend to serve as flexible scaffolds and bind multiple chromatin complexes [23]. One previous study using a RIP method showed that Snhg3 can interact with several chromatin-remodeling proteins including PRC2, JARID1B, RING1B, and SUV39H1, which can be characterized as chromatin “writers” (PRC2, SUV39H1), an “eraser” (JARID1B), and a “reader” (RING1B) [4]. However, we failed to identify these proteins in our RNA pull-down and mass spectrometry results. Additionally, there was no significant overlap in affected gene expression profiles from Snhg3 and its interacting chromatin remodeling proteins [4].

LncRNAs commonly regulate gene expression through their interactions with RNA-binding proteins (RBPs) [27]. Indeed, we found that Snhg3 binds several RBPs based on RNA pull-down and mass spectrometry results, including Hnrnpu, Hnrnpa2b1, Hnrnpa1, Hnrnpr, Hnrnph1, Nucleolin, and Pcbp2. Interestingly, some of these RBPs were reported to be associated with ESC maintenance. For example, Hnrnpu can activate Oct4 expression and is required for proper ESC proliferation [28, 29]. Further, knockdown of Hnrnpa2b1 was found to disrupt ESC proliferation via cell cycle arrest at the G0/G1 phase [30]. Moreover, Nucleolin can form a complex with lncRNAs or proteins in ESCs, which in turn maintains self-renewal and represses apoptosis [5, 31, 32]. Based on these data, we speculated that some RBPs such as Hnrnpu, Hnrnpa2b1, and Nucleolin could form complexes with Snhg3 to regulate specific classes of genes in mESCs. Further investigations are needed to confirm these interactions and potential functions.

Finally, Snhg3 may also play roles in induced pluripotent stem cell (iPSC) production and regulation. First developed in 2006, iPSCs are pluripotent stem cells reprogrammed from adult somatic cells by induced expression of transcription factors like Oct4/Sox2/Nanog/Lin28 or Oct4/Sox2/c-Myc/Klf4 [33, 34]. Obviating the ethical concern about the use of ESCs, iPSCs become attractive sources for organ regeneration and therapeutic application. Given that Snhg3 could form a feedback loop with Nanog and Oct4 in mESCs, it is possible that Snhg3 has a potential function in iPSCs. Further studies are required to validate the function of Snhg3 in iPSCs.

## Conclusions

In this study, we demonstrated the function of Snhg3 in mESC self-renewal, pluripotency, and early mouse embryonic development. Snhg3 was found to interact with core pluripotency factors and form a possible feedback loop to exert its function in mESCs. Our findings clarify the importance of long non-coding RNA in balancing the stemness and differentiation and indicate that it is essential for mouse early embryogenesis.

## Additional files

Additional file 1: **Figure S1.** Snhg3 affects apoptosis and proliferation in mESCs. a-b mESCs were transfected with siControl or siSnhg3 for 48 h, followed by Annexin V and PI staining. Representative results of flow cytometry (a) and the statistical analysis (b) showed that Snhg3 depletion resulted in less viable cells (Q3, Annexin V(−) and PI(−)) and more early apoptotic cells (Q4, Annexin V(+) and PI(−)).c The CCK-8 assay was used to evaluate the proliferation of Control or Snhg3 overexpressing mESCs for different time points. Data are presented as mean ± SD; *n* = 3, two-way ANOVA. **p < 0.01, ***p < 0.001 for all panels. (TIF 16312 kb)

Additional file 2: siRNAs and primer sequences. (DOCX 18 kb)

Additional file 3: Identified Snhg3 interacting proteins. (XLSX 26 kb)

## Data Availability

All data generated and/or analyzed in this study are included in this published article.

## Abbreviations

AP: Alkaline phosphatase
ceRNA: Competing endogenous RNA
ChIP: Chromatin immunoprecipitation
EB: Embryoid body
H3K27me3: Histone marker tri-methylation of histone 3 lysine 27
H3K4me4: Histone marker tri-methylation of histone 3 lysine 4
IF: Immunofluorescence staining
lncRNA: Long non-coding RNA
MEF: Mouse embryonic fibroblast
mESCs: Mouse embryonic stem cells
PI: Propidium iodide
qRT-PCR: Quantitative reverse transcription polymerase chain reaction
RBP: RNA-binding protein
RIP: RNA immunoprecipitation
Snhg3: Small nucleolar RNA host gene 3
WB: Western blotting

## References

[1] Young RA (2011). Control of the embryonic stem cell state. Cell..

[2] Morey L, Santanach A, Di Croce L (2015). Pluripotency and epigenetic factors in mouse embryonic stem cell fate regulation. Mol Cell Biol.

[3] Dinger ME, Amaral PP, Mercer TR, Pang KC, Bruce SJ, Gardiner BB (2008). Long noncoding RNAs in mouse embryonic stem cell pluripotency and differentiation. Genome Res.

[4] Guttman M, Donaghey J, Carey BW, Garber M, Grenier JK, Munson G (2011). lincRNAs act in the circuitry controlling pluripotency and differentiation. Nature..

[5] Lin N, Chang KY, Li Z, Gates K, Rana ZA, Dang J (2014). An evolutionarily conserved long noncoding RNA TUNA controls pluripotency and neural lineage commitment. Mol Cell.

[6] Derrien T, Johnson R, Bussotti G, Tanzer A, Djebali S, Tilgner H (2012). The GENCODE v7 catalog of human long noncoding RNAs: analysis of their gene structure, evolution, and expression. Genome Res.

[7] Guttman M, Amit I, Garber M, French C, Lin MF, Feldser D (2009). Chromatin signature reveals over a thousand highly conserved large non-coding RNAs in mammals. Nature..

[8] Ye Buqing, Liu Benyu, Yang Liuliu, Zhu Xiaoxiao, Zhang Dongdong, Wu Wei, Zhu Pingping, Wang Yanying, Wang Shuo, Xia Pengyan, Du Ying, Meng Shu, Huang Guanling, Wu Jiayi, Chen Runsheng, Tian Yong, Fan Zusen (2018). LncKdm2b controls self‐renewal of embryonic stem cells via activating expression of transcription factor Zbtb3. The EMBO Journal.

[9] Jain AK, Xi Y, McCarthy R, Allton K, Akdemir KC, Patel LR (2016). LncPRESS1 is a p53-regulated LncRNA that safeguards pluripotency by disrupting SIRT6-mediated de-acetylation of histone H3K56. Mol Cell.

[10] Xu C, Zhang Y, Wang Q, Xu Z, Jiang J, Gao Y (2016). Long non-coding RNA GAS5 controls human embryonic stem cell self-renewal by maintaining NODAL signalling. Nat Commun.

[11] Zhang T, Cao C, Wu D, Liu L (2016). SNHG3 correlates with malignant status and poor prognosis in hepatocellular carcinoma. Tumour Biol.

[12] Huang W, Tian Y, Dong S, Cha Y, Li J, Guo X (2017). The long non-coding RNA SNHG3 functions as a competing endogenous RNA to promote malignant development of colorectal cancer. Oncol Rep.

[13] Hong L, Chen W, Wu D, Wang Y (2018). Upregulation of SNHG3 expression associated with poor prognosis and enhances malignant progression of ovarian cancer. Cancer Biomark.

[14] Fei Fan, He Yongsheng, He Sen, He Zhongze, Wang Youyu, Wu Gang, Li Mengni (2018). LncRNA SNHG3 enhances the malignant progress of glioma through silencing KLF2 and p21. Bioscience Reports.

[15] Liu L, Ni J, He X (2018). Upregulation of the long noncoding RNA SNHG3 promotes lung adenocarcinoma proliferation. Dis Markers.

[16] Lu W, Fang L, Ouyang B, Zhang X, Zhan S, Feng X (2015). Actl6a protects embryonic stem cells from differentiating into primitive endoderm. Stem Cells.

[17] Lee DF, Su J, Sevilla A, Gingold J, Schaniel C, Lemischka IR (2012). Combining competition assays with genetic complementation strategies to dissect mouse embryonic stem cell self-renewal and pluripotency. Nat Protoc.

[18] Zhan S, Zhang X, Cao S, Huang J (2015). Benzo(a)pyrene disrupts mouse preimplantation embryo development. Fertil Steril.

[19] Keller GM (1995). In vitro differentiation of embryonic stem cells. Curr Opin Cell Biol.

[20] Liu X, Wang C, Liu W, Li J, Li C, Kou X (2016). Distinct features of H3K4me3 and H3K27me3 chromatin domains in pre-implantation embryos. Nature..

[21] Clouaire T, Webb S, Bird A (2014). Cfp1 is required for gene expression-dependent H3K4 trimethylation and H3K9 acetylation in embryonic stem cells. Genome Biol.

[22] Tu J, Tian G, Cheung HH, Wei W, Lee TL (2018). Gas5 is an essential lncRNA regulator for self-renewal and pluripotency of mouse embryonic stem cells and induced pluripotent stem cells. Stem Cell Res Ther.

[23] Hu W, Alvarez-Dominguez JR, Lodish HF (2012). Regulation of mammalian cell differentiation by long non-coding RNAs. EMBO Rep.

[24] Ghosal S, Das S, Chakrabarti J (2013). Long noncoding RNAs: new players in the molecular mechanism for maintenance and differentiation of pluripotent stem cells. Stem Cells Dev.

[25] Wang Y, Xu Z, Jiang J, Xu C, Kang J, Xiao L (2013). Endogenous miRNA sponge lincRNA-RoR regulates Oct4, Nanog, and Sox2 in human embryonic stem cell self-renewal. Dev Cell.

[26] Hoff AM, Alagaratnam S, Zhao S, Bruun J, Andrews PW, Lothe RA (2016). Identification of novel fusion genes in testicular germ cell tumors. Cancer Res.

[27] Sun X, Haider Ali MSS, Moran M (2017). The role of interactions of long non-coding RNAs and heterogeneous nuclear ribonucleoproteins in regulating cellular functions. Biochem J.

[28] Vizlin-Hodzic D, Johansson H, Ryme J, Simonsson T, Simonsson S (2011). SAF-A has a role in transcriptional regulation of Oct4 in ES cells through promoter binding. Cell Reprogram.

[29] Hasegawa Y, Brockdorff N, Kawano S, Tsutui K, Tsutui K, Nakagawa S (2010). The matrix protein hnRNP U is required for chromosomal localization of Xist RNA. Dev Cell.

[30] Choi HS, Lee HM, Jang YJ, Kim CH, Ryu CJ (2013). Heterogeneous nuclear ribonucleoprotein A2/B1 regulates the self-renewal and pluripotency of human embryonic stem cells via the control of the G1/S transition. Stem Cells.

[31] Li H, Wang B, Yang A, Lu R, Wang W, Zhou Y (2009). Ly-1 antibody reactive clone is an important nucleolar protein for control of self-renewal and differentiation in embryonic stem cells. Stem Cells.

[32] Yang A, Shi G, Zhou C, Lu R, Li H, Sun L (2011). Nucleolin maintains embryonic stem cell self-renewal by suppression of p53 protein-dependent pathway. J Biol Chem.

[33] Takahashi K, Tanabe K, Ohnuki M, Narita M, Ichisaka T, Tomoda K (2007). Induction of pluripotent stem cells from adult human fibroblasts by defined factors. Cell..

[34] Yu J, Vodyanik MA, Smuga-Otto K, Antosiewicz-Bourget J, Frane JL, Tian S (2007). Induced pluripotent stem cell lines derived from human somatic cells. Science..

