# Supplementary figures and images for: The long non-coding RNA Snhg3 is essential for mouse embryonic stem cell self-renewal and pluripotency
Source: Stem Cell Res Ther. 2019 May 31;10:157. doi: 10.1186/s13287-019-1270-5 (PMC6545032; doi:10.1186/s13287-019-1270-5)

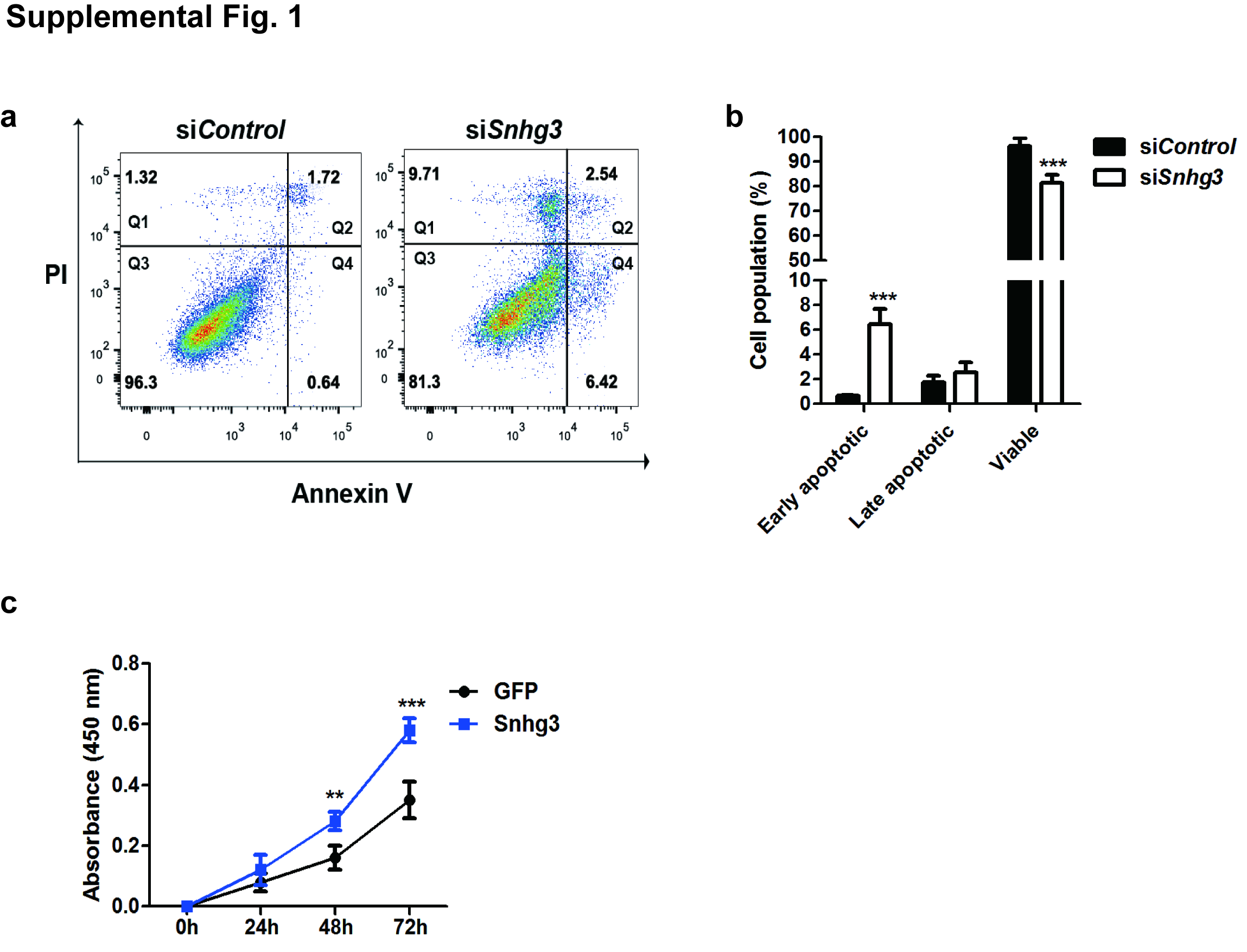

Supplement: Supplementary file 1 — Figure S1. Snhg3 affects apoptosis and proliferation in mESCs. a-b mESCs were transfected with siControl or siSnhg3 for 48 h, followed by Annexin V and PI staining. Representative results of flow cytometry (a) and the statistical analysis (b) showed that Snhg3 depletion resulted in less viable cells (Q3, Annexin V(−) and PI(−)) and more early apoptotic cells (Q4, Annexin V(+) and PI(−)).c The CCK-8 assay was used to evaluate the proliferation of Control or Snhg3 overexpressing mESCs for different time points. Data are presented as mean ± SD; n = 3, two-way ANOVA. **p < 0.01, ***p < 0.001 for all panels. (TIF 16312 kb) [file 13287_2019_1270_MOESM1_ESM.tif]
