# Supplementary material for: The long non-coding RNA Snhg3 is essential for mouse embryonic stem cell self-renewal and pluripotency
Source: Stem Cell Res Ther. 2019 May 31;10:157. doi: 10.1186/s13287-019-1270-5 (PMC6545032; doi:10.1186/s13287-019-1270-5)
Supplement: Supplementary file 2 — siRNAs and primer sequences. (DOCX 18 kb) [file 13287_2019_1270_MOESM2_ESM.docx]

**Table S1 List of siRNAs**

| **Name** | **Sequence (5’-3’)** |
| --- | --- |
| si*Control* | UUCUCCGAACGUGUCACGUTT |
| si*Snhg3-1* | GCUCUCUUGGUGUGCUUGUUCUUGA |
| si*Snhg3-2* | CCGGUCAAUGAUUUCAGGUACUUUG |
| si*Nanog* | GCCAACCUGUACUAUGUUUAA |
| si*Oct4* | GUGGACCUCAGGUUGGACUTT |

**Table S2 qRT-PCR primers**

| **Symbol** | **Forward Primer** | **Reverse Primer** |
| --- | --- | --- |
| *Snhg3* | CTCTCTTGGTGTGCTTGTTCT | CTAATGGCCGAGGCTGTAAC |
| *Gapdh* | TCCCACTCTTCCACCTTCGATGC | GGGTCTGGGATGGAAATTGTGAGG |
| *Nanog* | TTCTTGCTTACAAGGGTCTGC | AGAGGAAGGGCGAGGAGA |
| *Oct4* | GCAGGAGCACGAGTGGAAAGCAAC | CAAGGCCTCGAAGCGACAGATG |
| *Sox2* | GCGGAGTGGAAACTTTTGTCC | CGGGAAGCGTGTACTTATCCTT |
| *Klf4* | ATCCTTTCCAACTCGCTAACCC | CGGATCGGATAGCTGAAGCTG |
| *Tbx3* | AGATCCGGTTATCCCTGGGAC | CAGCAGCCCCCACTAACTG |
| *Esrrb* | GTCCCTCTCCGCGTTAGC | GGGGCAGGTTCGTCATTT |
| *Gata6* | ACAGCCCACTTCTGTGTTCCC | GTGGGTTGGTCACGTGGTACAG |
| *Gata4* | TTCCTGCTCGGACTTGGGAC | TTCCCAGGCAGGTGGAGAATAAG |
| *Foxa2* | GGCCCAGTCACGAACAAAGC | CCCAAAGTCTCCACTCAGCCTC |
| *Sox17* | TTCCAAGACTTGCCTAGCATC | CTTTATGGTGTGGGCCAAAG |
| *Mash1* | GCCACCAGAATGACTTCAGCAC | AAGGCAACCTATGGGAACCAAC |
| *Nestin* | CTGCAGGCCACTGAAAAGTTC | TCTGACTCTGTAGACCCTGCTTCTC |
| *Goosecoid* | AAACGCCGAGAAGTGGAACAAG | AAGGCAGGGTGTGTGCAAGTAG |
| *Branchyury* | CTCTAATGTCCTCCCTTGTTGCC | TGCAGATTGTCTTTGGCTACTTTG |
| *Sox1* | CCTCGGATCTCTGGTCAAGT | TACAGAGCCGGCAGTCATAC |
| *Cdk2* | CCTGCTTATCAATGCAGAGGG | TGCGGGTCACCATTTCAGC |
| *Cdk4* | ATGGCTGCCACTCGATATGAA | TCCTCCATTAGGAACTCTCACAC |
| *CyclinD1* | TCTACACTGACAACTCTATCCG | TAGCAGGAGAGGAAGTTGTTGG |
| *CyclinE1* | GTGGCTCCGACCTTTCAGTC | CACAGTCTTGTCAATCTTGGCA |
| *Xist* | GCCTCAAGAAGAAGGATTGC | GGGATTGTTTGTCCCTTTGG |
